# Critical role of SDF-1α-induced progenitor cell recruitment and macrophage VEGF production in the experimental corneal neovascularization

**Published:** 2011-08-10

**Authors:** Gaoqin Liu, Peirong Lu, Longbiao Li, Hui Jin, Xuefei He, Naofumi Mukaida, Xueguang Zhang

**Affiliations:** 1 Department of Ophthalmology, the First Affiliated Hospital of Soochow University, Suzhou, P.R. China; 2 Clinical Immunology Key Laboratory of Jiangsu Province, the First Affiliated Hospital of Soochow University, Suzhou, P.R. China; 3 Division of Molecular Bioregulation, Cancer Research Institute, Kanazawa University, Kanazawa, Japan

## Abstract

**Purpose:**

To address the roles of the stromal derived factor-1 (SDF-1) α in the course of experimental corneal neovascularization (CNV).

**Methods:**

CNV was induced by alkali injury and compared in SDF-1α- or vehicle-treated mice two weeks after injury. Angiogenic factor expression in the early phase after injury was quantified by reverse transcription polymerase chain reaction (RT-PCR). Progenitor cell, macrophage, and monocyte intracorneal accumulation in the early phase after injury was evaluated by flow cytometric analysis.

**Results:**

The mRNA expression of *SDF-1α* was augmented, together with infiltration of c-kit-positive progenitor cells in the corneas after the alkali injury. Compared with vehicle-treated mice, SDF-1α-treated mice exhibited enhanced CNV two weeks after injury, as evidenced by enlarged cluster of differentiation 31 (CD31)-positive areas. Concomitantly, the intracorneal infiltration of c-kit-positive progenitor cells but not F4/80+ macrophages or Ly-6G+ monocytes was significantly enhanced in SDF-1α-treated mice compared to vehicle-treated mice. SDF-1α enhanced vascular endothelial growth factor (*VEGF*) expression by murine peritoneal macrophages. Enhancement in intraocular *VEGF* expression was greater in SDF-1α-treated mice than in control mice after injury. Moreover, local administration of C-X-C chemokine receptor type 4 (CXCR4) antagonist after alkali injury reduced alkali-induced CNV.

**Conclusions:**

SDF-1α-treated mice exhibited enhanced alkali-induced CNV through enhanced intracorneal progenitor cell infiltration and increased *VEGF* expression by macrophages.

## Introduction

The cornea is characterized by the absence of blood vessels and hematopoietic cells including erythrocytes and leukocytes under physiologic conditions [1]. Corneal avascularity is required for optical clarity and maintenance of vision. Corneal neovascularization (CNV) arises from many causes including corneal infections, misuse of contact lens, chemical burn, and inflammation, and can lead to severe impaired vision [2-4]. Under most of these conditions, bone marrow (BM)-derived cells, neutrophils, and macrophages infiltrate the cornea. We previously proved that experimental CNV can occur independently of granulocyte infiltration [5]. Moreover, we observed that infiltrated macrophages exert complicated roles, by using different chemokine receptor and proinflammatory signals in the development of CNV [6-9].

The chemokine receptor, C-X-C chemokine receptor type 4 (CXCR4), was initially cloned as an orphan chemokine receptor and was found to be expressed on many different cell types such as monocytes, lymphocytes, hematopoietic and endothelial progenitor cells [10-13]. CXCR4 is activated by its single ligand, stromal-derived factor 1 (SDF-1/CXCL12), and mediates several different activities such as chemotaxis, adhesion, proliferation, survival, and, in some cells, apoptosis [14]. Activation of CXCR4 on lymphocytes and monocytes stimulates chemotaxis, resulting in recruitment to sites of immune and inflammatory reactions.

Accumulating evidence suggests that CXCR4/SDF-1α axis is involved in neovascularization. CXCR4 is also detected in endothelial cells [15]. Jin et al. [16] reported the involvement of SDF-1α in revascularization of ischemic hind limbs through recruitment of CXCR4^+^ hemangiocytes. Subcutaneous SDF-1α injection into mice induces infiltration of leukocytes such as monocytes/macrophages and small areas of neovascularization (NV) [17]. This enhanced NV may occur due to angiogenic factor expression by monocytes/macrophages. The CXCR4/SDF-1α axis plays a central role in the development of several types of ocular neovascularization including choroidal neovascularization, diabetic retinopathy, and oxygen-induced ischemic retinopathy [18-23]. Moreover, several independent studies suggest that SDF-1α may be responsible for abnormal vasculature in the posterior segment of the eye. BM-derived endothelial precursor cells (EPCs) have previously been shown to contribute to choroidal neovascularization by signaling through the SDF-1α/CXCR4 axis [24-26]. Moreover, mature vascular endothelial cells also express CXCR4 and its expression is upregulated by inflammatory cytokines and angiogenic factors including fibroblast growth factor (FGF) 2 and vascular endothelial growth factor (VEGF) [17,27-29]. Furthermore, Yu and colleagues [30] detected CXCR4 expression on human retinal microvascular endothelial cells associated their invasion and tubule formation.

The roles of the SDF-1α/CXCR4 axis in mediating corneal neovascularization subsequent to severe injury remain unclear. To further address the roles of CXCR4 signal in ocular neovascularization, the process of alkali-induced CNV was analyzed in SDF-1α- or CXCR4 antagonist-treated mice in comparison with control-treated mice. Here, we provided the definitive evidence to indicate the critical role of SDF-1α-induced progenitor cell recruitment and VEGF production by infiltrated macrophages in the experimental corneal neovascularization.

## Methods

### Reagents and antibodies

Rat anti-mouse F4/80 (clone A3–1) monoclonal antibody (mAb) was obtained from Serotec (Oxford, UK). Rat anti-mouse CD31 (MEC13.3), anti-mouse-Ly-6G (Clone IA8, catalog no. 551495) mAbs were purchased from BD PharMingen (San Diego, CA). Goat anti-mouse c-kit (AF1356) antibody and recombinant mouse SDF-1α (catalog no. 460-SD/CF) were supplied by R&D Systems (Minneapolis, MN). Goat anti-mouse VEGF (sc-1836) polyclonal antibodies and CXCR4 antagonist (AMD3100, SC-252367) were from Santa Cruz Biotechnology (Santa Cruz, CA).

### Mice

Specific pathogen-free 7 to 8 weeks old male BALB/c mice weighing 20 to 25 g were obtained from Shanghai SLAC Laboratory Animal Co. Ltd (Shanghai, China) and were kept in our animal facility under specific pathogen-free conditions. All animal experiments were done in accordance with the Guideline for the Care and Use of Laboratory Animals on the Chinese Medical Academy and the Soochow University Animal Care Committee, and with the ARVO Statement for the Use of Animals in Ophthalmic and Vision Research. Animals were kept in groups of 5 and fed regular laboratory chow and water ad libitum. A 12-h day and night cycle was maintained.

### Alkali-induced corneal injury model

Corneal injury was induced by placing a 2-mm filter disc saturated with 1N NaOH onto the left eye of the mouse for 45 s as previously described [5-9]. SDF-1α was dissolved in 0.2% sodium hyaluronate (Sigma-Aldrich, St. Louis, MO) immediately before topical application. In some experiments, the alkali-treated eyes received 5 μl of SDF-1α dissolved in 0.2% sodium hyaluronate at a concentration of 5 μg/ml, or 5 μl of 0.2% sodium hyaluronate as vehicle twice a day for 7 days immediately after the alkali injury. In another series of experiments, the eyes were treated with alkali for 45 s and received 5 μl of CXCR4 antagonist at a concentration of 100 μg/ml, or 5 μl of 0.2% sodium hyaluronate as vehicle twice a day for 7 days immediately after the alkali injury. At the indicated time intervals, mice were sacrificed, and whole eyes were removed. The eyes were snap-frozen in optimal cutting temperature (OCT) compound for histological analysis, or the corneas were removed and placed immediately into RNALate (Qiagen, Tokyo, Japan), and kept at −86 °C until total RNA extraction was performed. Each experiment was repeated at least three times.

### Biomicroscopic examination

Eyes were examined under a surgical microsystem (Leica MZ16, Wetzlar, Germany) 14 days after alkali injury by two independent observers with no prior knowledge of the experimental procedures, as described previously [6-9].

### Flow cytometrical analysis of intracorneally infiltrating progenitor cells, macrophages and monocytes

Mononuclear cells were isolated from corneas according to the procedure described previously with some modifications [6]. Briefly, at 4 days after the alkali injury, corneas were removed, were teased away with scissors, and were incubated at 37 °C for 30 min with constant shaking in the presence of 0.5 mg/ml collagenase type D (Roche Diagnostics, Mannheim, Germany). Cell suspensions were then passed over a nylon filter with 100-µm pore size. The resultant cells were further stained with goat anti-c-kit Ab following by staining with FITC-conjugated rat anti-goat IgG mAb. In another series of experiments, the resultant cells were stained with rat anti-mouse F4/80 or rat anti-mouse Ly-6G Ab followed by staining with PE-conjugated swine anti-rat IgG mAb. Fluorescence intensities were determined with the help of FACS Calibur (Becton Dickinson, Franklin Lakes, NJ), together with the samples stained with non-immunized goat or rat IgG (Sigma Aldrich) as an isotype control.

### Enumeration of corneal neovascularization

The fixed cryosections (8-µm thick) were stained using anti-CD31 mAb. The numbers and sizes of the CNV were determined as described previously [6-9], by an examiner with no previous knowledge of the experimental procedures. Briefly, images were captured with a digital camera and imported into Adobe Photoshop. Then, the numbers of neovascular tubes per mm^2^, and the proportions of CNV in the hot spots were determined using NIH Image analysis software version 1.62 (National Institutes of Health, Bethesda, MD). Most sections were taken from the central region of the cornea. The numbers and areas of corneal neovascularization were evaluated on at least two sections from each eye.

### Semi-quantitative reverse transcription (RT)-polymerase chain reaction (PCR)

Total RNAs were extracted from the corneas or cultured macrophages with the use of RNeasy Mini Kit (Qiagen, Tokyo, Japan). The resultant RNA preparations were further treated with RNase-free DNase (DNase) I (Life Technologies Inc., Gaithersburg, MD) to remove genomic DNA. Two μg of total RNAs were reverse-transcribed at 42 °C for 1 h in 20 μl of reaction mixture containing mouse Moloney leukemia virus reverse transcriptase and hexanucleotide random primers (Qiagen). Serially twofold diluted cDNA was amplified for β-actin (*Actb*) to estimate the amount of transcribed cDNA. Then, equal amounts of cDNA products were amplified for the target genes using the primers under the following conditions; denaturation at 94 °C for 2 min, followed by the optimal cycles of 30 s at 94 °C, 45 s at 55–58 °C, 1 min at 72 °C, and a final 10 min extension step at 72 °C. Sequences of the primers and PCR conditions were listed in Table 1. The amplified PCR products were fractionated on a 1.0% agarose gel and visualized by ethidium bromide staining. The band intensities were measured and their ratios to *Actb* were determined with the aid of NIH Image analysis software.

**Table 1:** Sequences of the primers used for reverse transcription polymerase chain reaction.

**Primers**	**Sequence (5´→3´)**	**Product size (bp)**	**Annealing temperature (ºC)**	**PCR cycles**
*ADAMTS-1*	(F) CAGTACCAGACCTTGTGCAGACCTT	299	58	37
	(R) CACACCTCACTGCTTACTGGTTTGA			
*SDF-1α*	(F) TGCCCCTGCCGGTTCTTCGAG	311	58	37
	(R) CTGTTGTTGTTCTTCAGCCGTGCAA			
*VEGF*	(F) CTGCTGTACCTCCACCATGCCAAGT	509	57	37
	(R) CTGCAAGTACGTTCGTTTAACTCA			
*TGF-β*	(F) CGGGGCGACCTGGGCACCATCCATGAC	405	57	37
	(R) CTGCTCCACCTTGGGCTTGCGACCCAC			
*MMP-2*	(F) GAGTTGGCAGTGCAATACCT	666	57	38
	(R) GCCATCCTTCTCAAAGTTGT			
*MMP-9*	(F) AGTTTGGTGTCGCGGAGCAC	754	57	37
	(R) TACATGAGCGCTTCCGGCAC			
*TNF-α*	(F) CAGCCTCTTCTCATTCCTGCTTGTG	511	58	36
	(R) CTGGAAGACTCCTCCCAGGTATAT			
*TSP-1*	(F) ACCAAAGCCTGCAAGAAAGA	311	57	37
	(R) ATGCCATTTCCACTGTAGCC			
*TSP-2*	(F) CAGAGTACTGGCGTCGGTCA	649	57	37
	(R) ATAAGATCGCAGCCCACATACAG			
*CXCR4*	(F) ATGTAGACACTGGCGGAAATGG	459	57	37
	(R) AGGTGGGGCGAAAGGAAAC			
*Actb*	(F) TGTGATGGTGGGAATGGGTCAG	514	55	25
	(R) TTTGATGTCACGCACGATTTCC			

All primers used were purchased from Genset Oligos (Kyoto, Japan). The amplification was performed using a GeneAmp® PCR System 9700 (Perkin-Elmer, Foster City, CA). In the table, "F" indicates forward primer and "R" indicates reverse primer.

### Western blot analysis

Corneal cell lysates were prepared from the indicated time interval after alkali injury. Protein samples were dissolved in Laemmli buffer, boiled for 3–4 min, and centrifuged for 2 min at 20,000× g to remove insoluble materials. Protein (30 μg) per lane was separated by sodium dodecyl sulfate (SDS)/PAGE (PAGE; 12%) and transferred to a 0.2 μm nitrocellulose membrane. The blocked membranes were probed overnight (4 °C) with goat anti-mouse VEGF antibodies (sc-1836, 1:100; Santa Cruz Biotechnology), and rabbit anti-mouse β-actin (N-21, 1:200; Santa Cruz Biotechnology). Subsequently, the membranes were incubated with horseradish peroxidase-conjugated secondary antibody, and immunoreactive bands were visualized using ECL reagent (Pierce). Immunoreactive bands corresponding to VEGF were quantified by Image J analysis and normalized to those of β-actin.

### Isolation and culture of murine peritoneal macrophages

Peritoneal macrophages were obtained from mice as described previously [6]. The cells were suspended in antibiotic-free RPMI medium containing 10% fetal bovine serum (FBS), and incubated in a humidified incubator at 37 °C in 5% CO_2_ in 6-well cell culture plates (Nalge Nunc International Corp., Naperville, IL). Two hours later, non-adherent cells were removed, and the medium was replaced. The cells were then stimulated with the indicated concentrations of murine SDF-1α for 12 h. Total RNAs were extracted from the cultured cells and subjected to RT–PCR as described above. In another series of experiments, for an immunocytochemical analysis of VEGF expression, murine macrophages were seeded onto the wells of a Lab-Tec chamber slide with eight wells (Nalge Nunc) at 5×10^4^ cells/well. After non-adherent cells were removed, the cells were stimulated with 100 μg/ml of murine SDF-1α for 12 h in a 37 °C incubator with 5% CO_2_ and then subjected to immunocytochemical study as previous described [31]. To test the expression of CXCR4 from the peritoneal macrophages by flow cytometrical analysis, cells were stained with rat anti-mouse F4/80 mAb and rabbit anti-mouse CXCR4 Ab followed by staining with FITC-conjugated goat anti-rabbit IgG mAb and phycoerythrin (PE)-conjugated swine anti-rat IgG mAb. Fluorescence intensities were determined with the help of FACS Calibur (Becton Dickinson), together with the samples stained with rat anti-mouse F4/80 mAb and non-immunized rabbit IgG as a control.

### Statistical analysis

The means and standard errors of the mean (SEM) were calculated for all parameters determined in the study. Data were analyzed statistically using one-way ANOVA (ANOVA), or two-tailed Student’s *t*-test. A value of p<0.05 was considered statistically significant.

## Results

### Intracorneal *SDF-1α* mRNA expression after alkali-induced corneal injury

**Figure 1:**
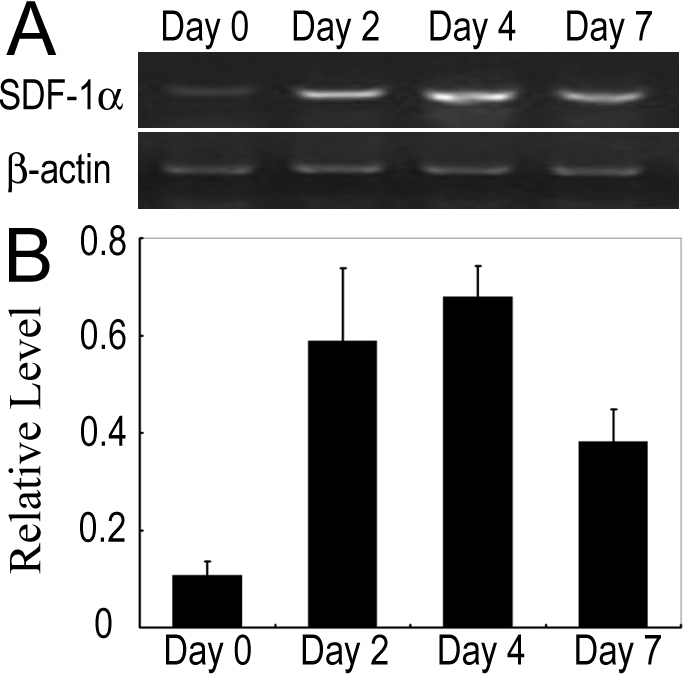
*SDF-1α* mRNA expression in corneas after alkali injury. **A**: Semi-quantitative RT–PCR to evaluate mRNA expression of *SDF-1α*. Corneas were harvested at the indicated time points, and five corneas at each time point were pooled to extract total RNAs. RT–PCR was performed using the obtained total RNAs. **B**: The ratios of *SDF-1α* to *Actb* mRNA were determined. All values represent mean±SEM of three to five independent measurements.

We first examined *SDF-1α* mRNA expression in corneas after alkali-induced corneal injury. *SDF-1α* mRNA was faintly detected in untreated eyes, but was markedly increased after alkali injury (Figure 1A,B). The enhanced intracorneal *SDF-1α* mRNA expression prompted us to investigate the possible involvement of the SDF-1α-CXCR4 interactions in alkali-induced CNV.

### Effects of SDF-1α on alkali-induced CNV

**Figure 2:**
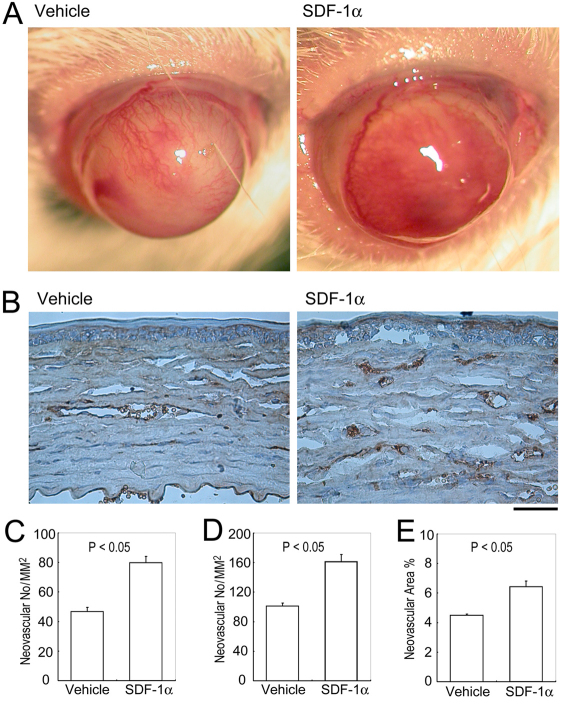
The effects of SDF-1α on Alkali injury-induced CNV. **A**: Macroscopic appearance of vehicle- or SDF-1α-treated BABL/c mouse eyes 2 weeks after alkali injury. **B**: Corneal tissues were obtained 2 weeks after injury from vehicle- or SDF-1α-treated BABL/c mice. Tissues were immunostained with anti-CD31 antibody, and representative results from five to eight animals are shown here. Original magnifications, 200×. Scale bar, 50 μm. **C**: CNV numbers per square millimeter in whole section. **D**: CNV numbers per square millimeter in hot spots. **E**: Proportions of CNV areas in hot spots were determined from the corneas obtained from vehicle- or SDF-1α-treated BABL/c mice 2 weeks after injury. Each value represents mean±SEM (n=5–8 animals). *, p<0.05 compared with vehicle-treated mice.

We next explored the effects of SDF-1α on alkali-induced CNV. CNV was macroscopically evident in mice 2 weeks after the injury, consistent with our previous reports [5-9]. Macroscopic inspection demonstrated that untreated corneas were avascular, but that alkali injury markedly increased the vascular areas in corneas to a larger extent in SDF-1α-treated mice than vechicle-treated ones (Figure 2A). Immunohistochemical analysis using anti-CD31 antibodies further revealed that CD31-positive areas were larger in SDF-1α-treated mice than in vehicle-treated ones (Figure 2B-E). These observations indicate that the SDF-1α-CXCR4 axis could promote alkali-induced CNV.

### Enhanced c-kit^+^ progenitor cell infiltration in the wound corneas in SDF-1α treated mice

**Figure 3:**
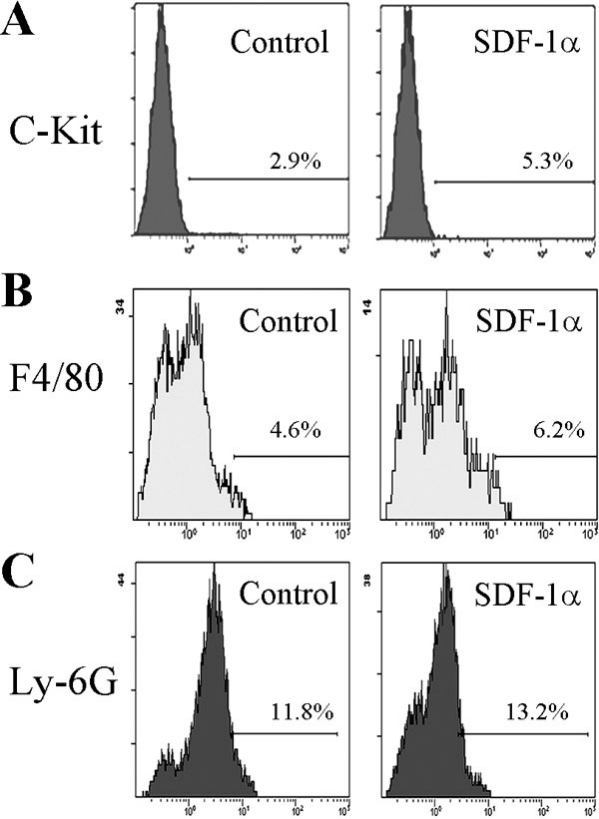
c-kit-, F4/80- or Ly-6G-positive cell numbers in cornea after alkali injury. Corneal tissues were obtained 4 days after injury from vehicle- or SDF-1α-treated BABL/c mice, and the tissues from 7 to 8 mice were combined and were subjected to analysis using a flow cytometer after being immunostained with anti-c-kit, anti-F4/80, or anti-Ly-6G antibody. Isotype IgG derived from the same species of the test antibody was used as negative control. Representative results from three to four tests of intracorneal infitlration of c-kit- (**A**), F4/80- (**B**), or Ly-6G-positive cells (**C**) from either vehicle- (left plot) or SDF-1α-treated mice (right plot) are shown.

We previously observed that Ly-6G-positive granulocytes and F4/80-positive macrophages infiltrated the injured cornea, reaching their peak levels two to four days after the injury in mice [6]. Ly-6G-positive granulocytes and F4/80-positive macrophages infiltrated corneas to a similar extent in SDF-1α- and vehicle-treated mice after the injury (Figure 3, Table 2). On the contrary, c-kit-positive progenitor cell infiltration was markedly augmented in SDF-1α-treated mice compared with vehicle-treated ones (Figure 3, Table 2). These observations would indicate that SDF-1α treatment further augmented alkali-induced infiltration of c-kit-positive progenitor cells and that macrophages infiltrated as a consequence of injury rather than as a consequence of topical administration SDF-1α.

**Table 2:** SDF-1α topical administration on intracorenal cell infiltration.

**Target protein**	**Control**	**SDF-1α**	**p value (F test)**
C-Kit	3.08±0.37	9.85±1.58	<0.05
F4/80	5.20±0.38	7.73±0.49	>0.05
Ly-6G	12.37±1.36	12.97±1.32	>0.05

### Enhanced *VEGF* expression in SDF-1α-treated mice after alkali injury

**Figure 4:**
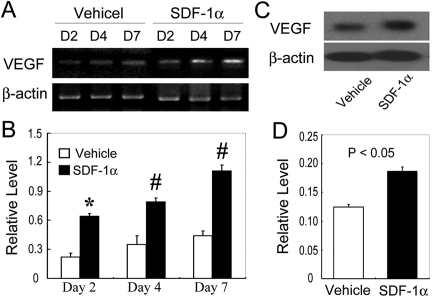
*VEGF* expression in the injured corneas of mice. **A**: Semi-quantitative RT–PCR to evaluate mRNA expression of *VEGF*. Corneas were harvested at the indicated time points, and five corneas at each time point were pooled to extract total RNAs. RT–PCR was performed using the obtained total RNAs. Representative results from three independent are shown here. **B**: The ratios of *VEGF* to *Actb* mRNA were determined on vehicle- (open bars) and SDF-1α-treated BABL/c mice (black bars). All values represent mean±SEM of three to five independent measurements. **C**: Protein extracts were obtained and subjected to western blotting analysis. Representative results from three independent experiments are shown here. **D**: Ratios of VEGF to β-actin protein bands of vehicle- (open bars) and SDF-1α-treated mice (black bars) were determined as described in Methods. All values represent mean±SEM (n=5–8 animals). *, p<0.05 and ^#^, p<0.01 compared with vehicle-treated mice.

The balance between angiogenic and anti-angiogenic factors determines the outcome of angiogenesis processes in various situations. Hence, we examined the mRNA expression of angiogenic and anti-angiogenic factors in corneas after alkali injury. Alkali injury enhanced mRNA expression of various angiogenic associated factors, including *VEGF*, transforming growth factor (*TGF*)-β, matrix metalloproteinase (*MMP*)-2, *MMP-9*, tumor necrosis factor (*TNF*)-α, a disintegrin and membranous thrombospondin (*ADAMTS*)-1, thrombospondin (*TSP*)-1, and *TSP-2*, similarly as we described previously. Among these factors that we examined, only *VEGF* mRNA expression was augmented to a larger extent in SDF-1α-treated mice than vehicle-treated ones in the early phase after injury (Figure 4A,B). Consistently, intraocular VEGF protein expression was increased to a larger extent in SDF-1α-treated mice compared to vehicle-treated ones (Figure 4C,D). Thus, exogenous administration of SDF-1α augmented the expression of a pro-angiogenic factor, VEGF, thereby tipping the balance to promote angiogenesis.

### Enhanced *VEGF* expression by murine macrophages with SDF-1α stimulation

**Figure 5:**
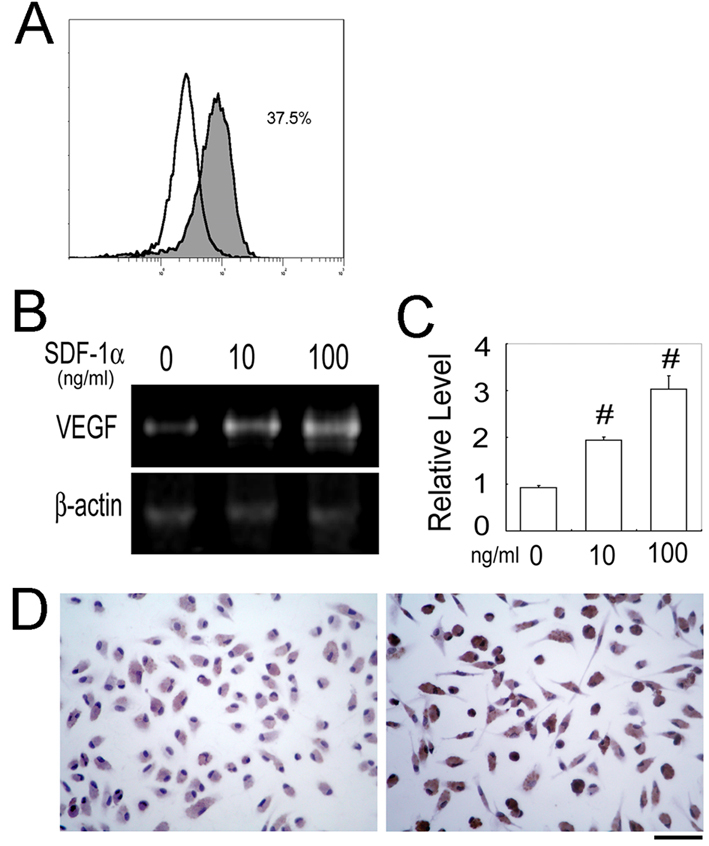
SDF-1α-induced VEGF production by peritoneal macrophages. **A**: CXCR4 expression on F4/80-positive murine peritoneal macrophages was determined by a flow cytometric analysis. Purified mononuclear cells were stained with rat anti-mouse F4/80 mAb and rabbit anti-CXCR4 Abs (filled histogram) or rat anti-mouse F4/80 mAb and non-immunized rabbit IgG (open heavy-lined histogram) as a control followed by staining with FITC-conjugated goat anti-rabbit IgG and PE-conjugated swine anti-rat IgG. A representative result from three independent experiments is shown. **B**: Peritoneal macrophages from WT mice were incubated with the indicated concentrations of SDF-1α for 12 h. Quantitative RT–PCR was performed on total RNAs extracted from the macrophages as described in Methods. Representative results from 3 independent experiments are shown here. **C**: *VEGF* mRNA levels were determined and normalized to *Actb* mRNA levels. Each value represents the mean and SEM (n=3). ^#^, p<0.01 compared with untreated. **D**: VEGF protein expression was detected by immunocytochemical analysis using anti-VEGF Abs as described in Methods. Representative results from three independent experiments are shown. Original magnification, 400×. Scale bar, 50 μm.

To delineate the roles of *CXCR4* expression on macrophages, we next examined the effects of SDF-1α on the expression of *VEGF* by macrophages. We detected *CXCR4* mRNA on murine peritoneal macrophages by RT–PCR (data not shown) and flow cytometric analysis detected CXCR4 expression on 37.5%–60.7% of F4/80-positive cells (Figure 5A). SDF-1α markedly enhanced *VEGF* mRNA expression by peritoneal macrophages in a dose-dependent manner (Figure 5B,C). Consistently, SDF-1α augmented VEGF protein expression by macrophages (Figure 5D). These observations would indicate that SDF-1α can induce macrophages to express a potent angiogenic molecule, VEGF, through the interaction with CXCR4.

### Topical administration of CXCR4 antagonist attenuated alkali injury induced corneal neovascularization in WT mice

**Figure 6:**
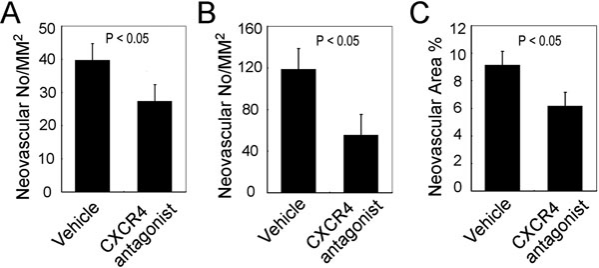
The effects of CXCR4 antagonist on alkali-induced CNV. CNV numbers per square millimeter in whole section (A), CNV numbers per square millimeter in hot spots (B), and percentage CNV areas in hot spots (C) were determined from the corneas obtained from vehicle- or CXCR4 antagonist-treated BABL/c mice 2 weeks after injury. Each value represents mean±SEM (n=5–8 animals). p<0.05 compared with vehicle-treated mice.

Finally, we examined the effects of topically-applied CXCR4 antagonist on alkali-induced CNV. Topical administration of CXCR4 antagonist in the early phase from day 1 to day 7 after alkali injury significantly attenuated the alkali-induced CNV compared to those of vehicle treatment (Figure 6). These results further suggest that the crucial involvement of the endogenously produced SDF-1α in alkali-induced CNV.

## Discussion

SDF-1α is a member of the CXC chemokine subfamily and is presumed to be involved in BM progenitor cells migration [32]. The reported contribution of BM-derived endothelial progenitor cells to the choroidal neovascularization [24-26,33] prompted us to speculate that SDF-1α may enhance CNV by recruiting progenitor cells to the injured corneas. Indeed, we observed increased intracorneal SDF-1α expression after alkali injury, and local administration of SDF-1α enhanced intracorneal c-kit-positive progenitor cell infiltration. Accumulating evidence indicates that BM-derived c-kit-positive cells can differentiate into endothelial cells, and enhance neovascularization [34]. Thus, it is reasonable to speculate that SDF-1α enhanced intracorneal c-kit-positive cell infiltration, thereby augmenting alkali-induced CNV.

Cornea avascularity is maintained by the balance between angiogenic and anti-angiogenic factor expression [2-4,35,36]. Corneal wounding enhanced predominantly the expression of angiogenic factors such as VEGF and basic fibroblast growth factor (bFGF) [37], and skewed the balance toward pro-angiogenic milieu, thereby causing CNV [38,39]. We observed that the expression of a potent angiogenic factor, VEGF, in alkali-injured cornea was enhanced to a larger extent when the mice were treated with SDF-1α, consistent with the previous report that the CXCR4/SDF-1α axis can induce Akt phosphorylation and eventually augment VEGF expression at both the mRNA and protein levels, thereby promoting VEGF-mediated tumor angiogenesis [40]. Moreover, accumulating evidence indicates that VEGF can stimulate SDF-1α-induced angiogenesis [17,22,41], by enhancing recruitment of CXCR4-expressing BM-derived progenitor cells [42-44]. Thus, our present observation raised the possibility that SDF-1α and VEGF form a vicious cycle, promoting alkali-induced CNV, particularly in its early phase.

Accumulating evidence indicates that BM-derived progenitor cells (EPCs) can differentiate into endothelial cells and contribute to the choroidal neovascularization [24-26]. It is also likely that EPCs can contribute to corneal neovascularization. The CXCR4-SDF-1α axis can control the differentiation of EPCs to endothelial cells [45,46]. Moreover, human retinal microvascular endothelial cells express *CXCR4* and exhibit tube formation and migration in response to SDF-1α [30,47]. These observations suggest that SDF-1α can induce EPC infiltration into cornea, its differentiation into endothelial cells, and affect the functions of the resultant endothelial cells in corneal neovascularization. Because VEGF can increase *CXCR4* expression on endothelial cells [17,48], VEGF can indirectly enhance neovascularization through interacting the CXCR4/SDF-1α axis in addition to its direct effects on neovascularization. These findings further suggest the potential role of the interactions between SDF-1α and VEGF on CNV.

Several lines of evidence indicate that macrophages can be pro-angiogenic by producing angiogenic factors in ocular neovascularization [49-53]. We assumed that SDF-1α may promote neovascularization by inducing macrophage recruitment. In contrast to our expectation, topical SDF-1α administration failed to enhance alkali injury-induced intracorneal F4/80-positive macrophage/monocyte infiltration despite CXCR4 mRNA and protein expression by a substantial proportion of monocytes/macrophages. The failure of SDF-1α to increase macrophage infiltration after injury could be due to saturation of the CXCR4s on macrophages by their ligand. Alternatively, full signaling activation may occur if only some of the receptors are occupied after alkali injury. Nevertheless, we observed that SDF-1α induced murine peritoneal macrophages to produce VEGF. Thus, the SDF-1α-CXCR4 axis can be crucial to VEGF production by CXCR4-expressing macrophages but not monocyte/macrophage infiltration into cornea. Moreover, CXCR4-positive macrophages can be pro-angiogenic similarly to CCR2-expressing macrophages as we previously reported [9].

The administration of CXCR4 antagonist remarkably attenuated alkali-induced CNV, indicating the crucial involvement of endogenously produced SDF-1α in this process. Age-related macular degeneration (AMD), an ocular disease with choroidal neovascularization, is a leading cause of blindness worldwide. Lee and colleagues reported that inhibition of SDF-1α can reduce laser-induced choroidal neovascularization, a murine AMD model [23]. Guerin and colleagues observed that SDF-1α, which may be produced by the RPE, could play a role in human choroidal neovascularization [54]. Furthermore, macrophages are found to be present around neovascular areas in wet AMD patients [55]. Considering that the SDF-1α-CXCR4 interactions can regulate pro-angiogenic activities of macrophages, it is tempting to speculate that the axis has a crucial role in AMD. If so, blockade of this axis can be effective for treatment and/or prevention of AMD.
